# Specific Activation of *K-Ras^G12D^* Allele in the Bladder Urothelium Results in Lung Alveolar and Vascular Defects

**DOI:** 10.1371/journal.pone.0095888

**Published:** 2014-04-23

**Authors:** Francisco Ayala de la Peña, Keizo Kanasaki, Megumi Kanasaki, Sylvia Vong, Carlota Rovira, Raghu Kalluri

**Affiliations:** 1 Division of Matrix Biology/Department of Medicine, Beth Israel Deaconess Medical Center and Harvard Medical School, Boston, Massachusetts, United States of America; 2 Department of Pathology, Hospital Sant Joan de Deu, Esplugues de Llobregat, Barcelona, Spain; 3 Department of Cancer Biology, Metastasis Research Center, University of Texas M. D. Anderson Cancer Center, Houston, Texas, United States of America; University of Giessen Lung Center, Germany

## Abstract

*K-ras* is essential for embryogenesis and its mutations are involved in human developmental syndromes and cancer. To determine the consequences of *K-ras* activation in urothelium, we used uroplakin-II (UPK II) promoter driven Cre recombinase mice and generated mice with mutated *Kras^G12D^* allele in the urothelium (*UPK II-Cre;LSL-K-ras^G12D^*). The *UPK II-Cre;LSL-K-ras^G12D^* mice died neonatally due to lung morphogenesis defects consisting of simplification with enlargement of terminal air spaces and dysmorphic pulmonary vasculature. A significant alteration in epithelial and vascular basement membranes, together with fragmentation of laminin, points to extracellular matrix degradation as the causative mechanism of alveolar and vascular defects. Our data also suggest that altered protease activity in amniotic fluid might be associated with matrix defects in lung of *UPK II-Cre;LSL-K-ras^G12^*. These defects resemble those observed in early stage human neonatal bronchopulmonary dysplasia (BPD), although the relevance of this new mouse model for BPD study needs further investigation.

## Introduction

Ras proteins, a super-family of cell membrane GTPases, are key regulators of pathways involved in cell survival, proliferation and differentiation [1]. Activating somatic point mutations of *Ras* are very frequent in cancer [2]. Some recent studies have demonstrated that germline *Ras* mutations play a role in a sub-group of developmental disorders collectively known as neuro-facial-cardio-cutaneous syndromes [3]. Additionally, mouse models have shown an essential role for K-ras in embryogenesis [4], [5]. Oncogenic *Ras* somatic mutations stabilize the GTP-bound conformation of the protein, constitutively activating their effector pathways and their biological effects being dependent upon the type of mutation and the cellular context [6]. Mutations found in developmental disorders [7] are also activating mutations, and in fact the widespread expression of mutated *K-ras^G12D^* allele in the embryos is lethal due to impaired placental function [8] including heart and brain development defects [9]. However, its conditional expression in epithelia causes hyperplasia and dysplasia, sometimes leading to carcinomas [9].

Urothelial expression of *H-ras* results in superficial papillary bladder neoplasms in the adult mice [10], [11]. The role of *K-ras* in the urothelial cells is less well understood [12], although some recent works have shown its participation in bladder hyperplasia [13] and the implication of RAS/MAPK signaling in urothelial carcinoma in situ [14]. We here describe a mouse with conditional activation of *K-ras^G12D^* in urothelium (*uroplakin(UPK)II-Cre;LSL-K-ras^G12D^*). These mice die at neonatal stage due to lung developmental defects resembling human neonatal bronchopulmonary dysplasia (BPD), a chronic neonatal lung disease associated with premature birth and currently considered more of a developmental disorder with impairment of lung alveolarization [15], [16].

## Materials and Methods

### Ethics Statement

The animal experiments were conducted in accordance with the recommendations in the Guide for the Care an Use of Laboratory Animals of the National Research Council. The protocol was approved by the Institutional Animal Care and Use Committee of the Beth Israel Deaconess Medical Center (Protocol number: 120–2008). Pregnant mice were euthanized by cervical dislocation. Fetuses extracted from the uterus and neonate mice were euthanized by decapitation. All efforts were made to minimize suffering.

### Gene constructs and generation of transgenic mice

We generated a transgenic mouse that expresses the Cre recombinase enzyme in bladder epithelium by utilizing the urothelium-specific promoter uroplakin (UPK)-2 (3.6 Kb), as previously reported [17]. With a PCR-based approach we cloned mouse *UPK II* promoter (Accession number: EF467361) from BAC (bacterial artificial chromosome) clone RP24-308H8. During the cloning process we noticed that a portion of the reported sequence of *UPK II* promoter region (Accession number: U14421) was miscloned because of opposite insertion of two *SacI* restriction enzyme sites (from −1262 to −2805 from Exon1) in *UPK II* promoter. Microinjection of the purified constructs in the pronuclei of fertilized eggs from C57/BL mice was performed for generation of transgenic mice *UPK II-Cre* at Beth Israel Deaconess Medical Center transgenic facility.

### Mouse strains and experiments with mice

For confirmation of urothelium-restricted expression of *UPK II*, a reporter strain was generated by interbreeding the *UPK II-Cre* strain to *ROSA-Stop -Lox-YFP* mice. Post-natal day 1 (P1) mice were sacrificed by decapitation and tissues were fixed in 4% PFA overnight, then in 20% sucrose and finally included in OCT and snap frozen. The whole lung, ureter and bladder and representative samples from placenta and yolk sac were evaluated for YFP expression. A *Lox-Stop-Lox (LSL)-K-ras^G12D^* mouse strain (Balb-C background), obtained from Jackson Laboratories, was bred to *UPK II-Cre* mice (C57BL background). The finding of a vaginal plug was considered as day 0.5 of pregnancy. Early lethality (day P1) appeared in the first litter of *UPK II-Cre;LSL-K-ras^G12D^* mice. Observational studies were then performed in two additional litters, with observation of pregnant mother each two hours and hourly litter observation of breathing and activity from birth, thereby ensuring prompt retrieval of non-surviving neonates and adequate tissue processing of tissues. After whole lethality was confirmed, all newborn (P1) mice of each litter were sacrificed by decapitation in the first 2-6 hours of life, before spontaneous death of any of the pups. Embryos (E14.5, E17.5 and E19.5) were collected with the amniotic sac intact and AF volume was determined. Lungs and the rest of organs were snap-frozen or fixed in 4% paraformaldehyde. The term ‘controls’ refers to littermates Cre recombinase (-) mice. Mice were maintained at the Beth Israel Deaconess Medical Center animal facility under standard conditions.

### Histological techniques

Paraformaldehyde-fixed, paraffin-embedded 5 µm sections were treated with 10 mM citrate buffer for antigen retrieval and standard immunohistochemical techniques. Primary antibodies: anti-pan-laminin (1∶250; Sigma-Aldrich, St. Louis, MO) and anti-CD34 (1∶50; Abcam, Cambridge, MA). Biotinylated secondary antibodies (1∶200) and Vectastain ABC kit were used according to manufacturer's instructions (Vector Laboratories, Burlingame, CA). 5′-Bromo-2′-doxyuridine (Sigma-Aldrich, St. Louis, MO) was injected intraperitoneally (10 µg/gr. body weight) 2 hours before sacrifice and tissues were labeled using the BrdU Labeling and detection Kit III (Roche, Indianapolis, IN). For BrdU quantification, we analyzed digital images by counting the number of BrdU labeled nuclei in each 200 µm segment of urothelium. For immunofluorescence, 5 µm frozen sections were fixed in acetone at −20°C. Primary antibodies: anti-CD31 (1∶50; BD Biosciences Pharmingen, Sparks, MD), anti-CD34 (1∶50; Abcam, Cambridge, MA), anti-laminin (1∶100, Sigma-Aldrich, St. Louis, MO), anti-laminin β1 (1∶50; Santa Cruz Biotechnology, Santa Cruz, CA), anti-collagen IV α-1 (1∶100; Abcam, Cambridge, MA), anti-nidogen/entactin (1∶200; Millipore, Billerica, MA), anti-E-cadherin (1∶100; Santa Cruz Biotechnology, Santa Cruz, CA), anti-VEGF (10 µg/ml; Thermo Fisher Sci, Rockford, IL), anti-MMP-1 (1∶100; EMD Biosciences, Gibbstown, NJ), anti-MMP-3 (1∶100; EMD Biosciences, Gibbstown, NJ), and anti-MMP-13 (1∶100; EMD Biosciences, Gibbstown, NJ). Sections were subsequently labeled with fluorescein- or rhodamine-conjugated secondary antibodies (Jackson Immunoresearch, West Grove, PA) and nuclei counterstained with DAPI. Quantification of lung terminal air-space area was performed, after exclusion of large vessels or bronchi, with the Image J software (NIH, Bethesda, MD); both total and mean air space were normalized to lung surface and expressed as a ratio to control lung.

### Genotyping

Genotype of transgenic mice was determined by PCR in genomic DNA isolated from mice tails. To exclude Cre-mediated excision of the Stop-cassette in lung, we performed specific PCR of DNA isolated from lung, placenta and bladder using the Qiagen DNAeasy Blood&tissue kit (Qiagen, Valencia, CA). Sex was genotyped as reported [18].

### Amniotic fluid determinations

Lactate in AF samples was measured with the Lactate Assay Kit (Eton Bioscience Inc, Cambridge, MA) according to manufacturer's instructions. VEGF and sFLT1 were measured in AF samples with VEGF ELISA kit mouse (EMD Biosciences, Gibbstown, NJ) and Quantikine Mouse sVEGF R1 kit (R&D Systems, Minneapolis, MN).

### SDS-PAGE, Western Blot (WB) and zymography

Samples (1 µl of amniotic fluid samples or purified protein from lung or bladder) were denatured with SDS sample buffer. Samples for E-cadherin WB were prepared with NP-40 Lysis Buffer (Boston Bioproducts, Boston, MA). Primary antibodies: anti-laminin β1 (1∶500; Millipore, Billerica, MA), anti-E-cadherin (1∶1000; BD Biosciences Pharmingen, Sparks, MD), anti-MMP-1 (1∶1000; EMD Biosciences, Gibbstown, NJ), anti-MMP-2 (1∶250; Santa Cruz Biotechnology, Santa Cruz, CA), anti-MMP-3 (1∶500; Sigma-Aldrich, St. Louis, MO), anti-MT1-MMP (1∶500; EMD Biosciences, Gibbstown, NJ) and anti-actin (1∶1000; Sigma-Aldrich, St. Louis, MO). After incubation with HRP-conjugated secondary antibodies (Sigma-Aldrich, St. Louis, MO), an enhanced chemiluminescence detection system (Pierce Biotechnology, Rockford, IL) was used for development. For zymography, 10% SDS-PAGE gels were copolymerized with gelatin (0.1 mg/L) or casein (1 mg/L) (Sigma-Aldrich, St. Louis, MO). Between 1 and 5 µl of amniotic fluid were used for zymography, which was performed as previously described [19].

### Statistical analysis

Data are expressed as mean ± SE. Statistical analysis was performed using SPSS version 15.0 (SPSS Inc.). Tests used were non-parametric Mann-Whitney (unpaired, two-tailed), and, when appropriate, Student's T-test. Statistical significance was established as *P*≤0.05.

## Results

### UPK II-Cre expression is restricted to urothelium

**Figure 1:**
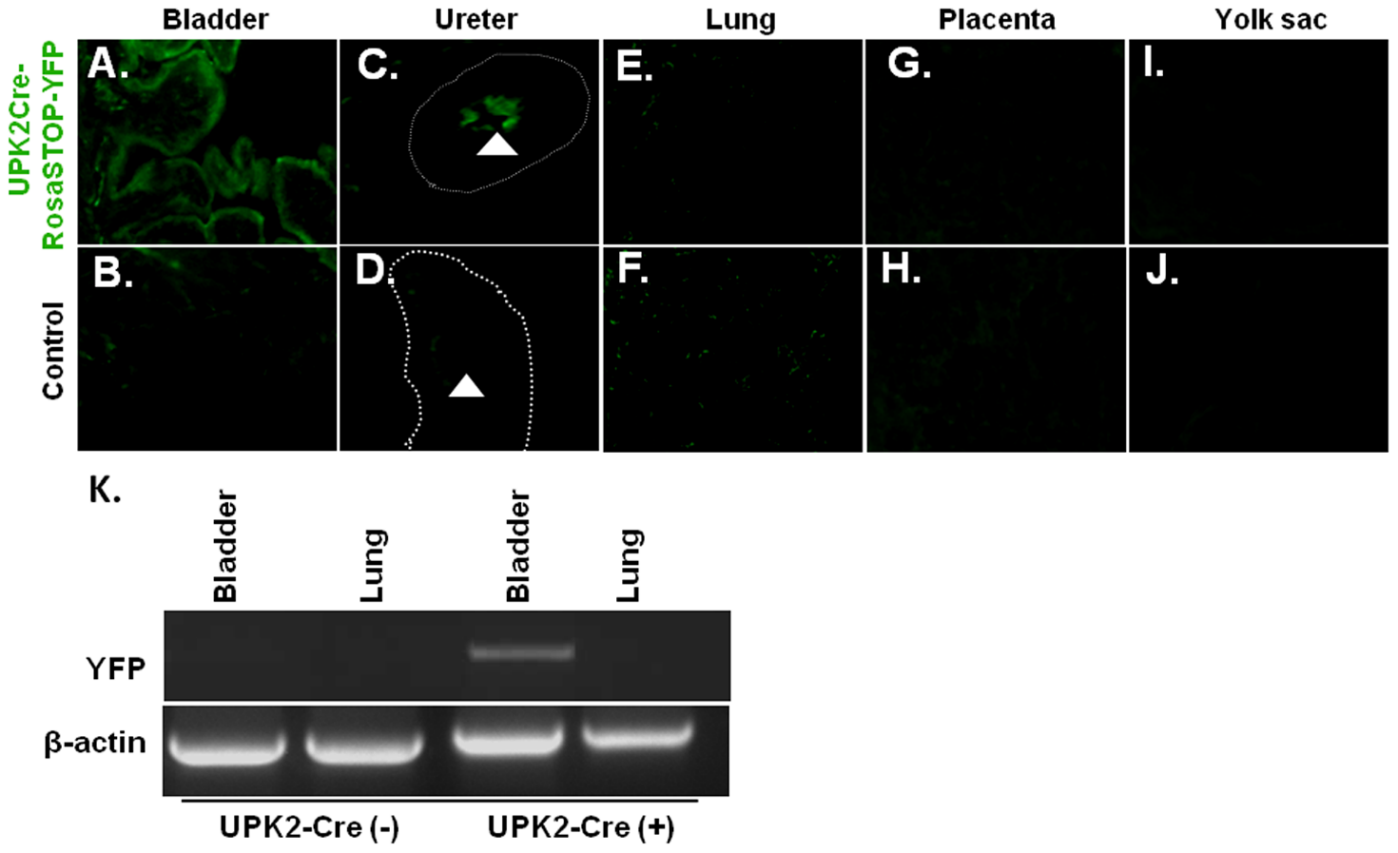
Characterization of the *UPK II-Cre;Rosa-Stop-YFP* reporter mice. A–J: *UPK II-Cre;Rosa-Stop-YFP^+/+^* reporter mice reveal fluorescence only in the bladder urothelium (x200) (**A,B**) and ureter urothelium (x200) (day 1, P1) (white arrow) (**C,D**), but not in lung (x200) (P1) (**E,F**), placenta (x200) (E19.5) (**G,H**) or yolk sac (x200) (E19.5) (**I,J**) among other negative tissues. **K**: PCR for recombinant UPK II-YFP was only positive in *UPK II-Cre;Rosa-Stop-YFP^+/+^* bladder (E19.5).

A transgenic mouse that expressed the Cre recombinase enzyme in bladder epithelium was generated by utilizing the urothelium specific promoter uroplakin II (UPK II). To confirm urothelium-restricted expression of the *UPK II-Cre* mice, a reporter strain was obtained by breeding the *UPK II-Cre* with *Rosa-R26R-YFP* floxed mice. When compared to control mice, YFP expression at both day P1 and E17.5 was only detected in the urothelial cells of bladder (Figure 1A–B) and the ureter (Figure 1C–D) of *UPK II-Cre;Rosa-Stop-YFP* ^+/+^, but not in lung (Figure 1E–F). YFP expression was not detected in the placenta or the yolk sac (Figure 1G–J) at E17.5 and E19.5. YFP expression was not only confined to the umbrella cell layer, but also detected in the basal and suprabasal layers of the urothelium [20]. Absence of Cre-mediated excision of the Stop cassette in lung was further confirmed by PCR for the recombined YFP gene, when compared to the bladder DNA from *UPK II-Cre;Rosa-Stop-YFP* mice at E19.5 (Figure 1K) and P1.

### Early lethality in UPK II-Cre;K-ras^G12D^ mice

**Figure 2:**
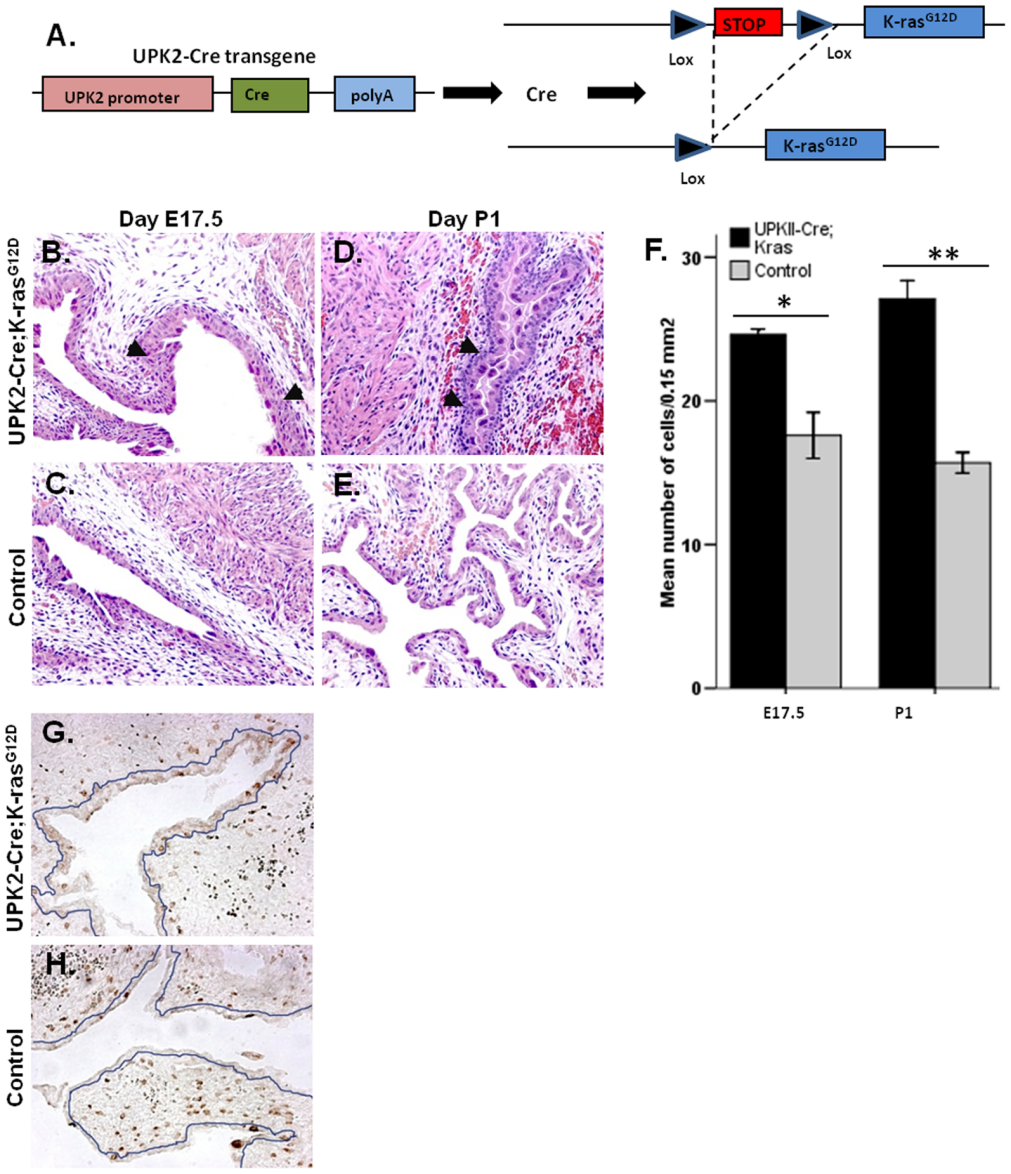
Urothelial hyperplasia in the *UPK II-Cre;LSL-K-ras^G12D^* mice. A: Urothelial-restricted expression of K-ras^G12D^. **B–E**: H&E analysis of bladders (X200) reveals a hyperplastic urothelium at E17.5 (**B,C**) and P1 (**D,E**) (black arrows). **F**: Differences in urothelial cellularity (cells/0.15 mm^2^) between *UPK II-Cre;LSL-K-ras^G12D^* mice and controls were significant both at E17.5 (n = 2/group; *, *P* = 0.05) and P1 (n>6/group; **, *P*<0.0001); bars, SEM. **G–H**: BRDU staining of bladder (X200) showing a higher proliferation in E17.5 *UPK II-Cre;LSL-K-ras^G12D^* mice (urothelium limit is marked with a blue line). The mean number of BrdU positive nuclei/200 µm of urothelium was significantly higher than in controls (n = 10; 6±2 positive nuclei/200 µm vs 1.33±0.81; *P* = 0.01).

Heterozygous mating of *UPK II-Cre* and *K-ras^G12D^* mice (Figure 2A) generated an average of 9.75 (range: 7–12) mice/litter. Sex ratio was well balanced (59.45% females, 40.55% males; χ^2^ test, *P* = 0.32). The 8 litters evaluated included 33 mice with the genotype *UPK II-Cre*+/K-ras^G12D^ (*UPK II-Cre;LSL-K-ras^G12D^*), confining to the expected Mendelian ratio. After observation of three double positive litters confirmed a complete lethality in the first 12 hours after birth (18/18 vs. 0/15; χ^2^ test, *P*<0.001), the rest of litters were sacrificed within 6 hours after birth and further evaluated. Mice with the *UPK II-Cre;LSL-K-ras^G12D^* genotype exhibited significantly lower weight (*UPK II-Cre;LSL-K-ras^G12D^* vs. control: 1.312±0.208 gr. vs. 1.419±0.096 gr.; *P* = 0.04) and had minimal mobility and activity.

### Urothelial expression of K-ras^G12D^ is associated with bladder epithelial hyperplasia

*K-ras^G12D^* expression induces proliferation and hyperplasia in epithelial cells [9]. When compared to the control mice, urothelium from *UPK II-Cre;LSL-K-ras^G12D^* mice showed a significant absolute increase in cell number, consistent with hyperplasia, both at E17.5 (Figure 2B–C) and P1 (Figure 2D–F). BrdU staining at E17.5 revealed increased urothelial proliferation in *UPK II-Cre;LSL-K-ras^G12D^* mice when compared to control mice (6+2 positive nuclei/200 µm vs. 1.33+0.81; *P* = 0.01) (Figure 2G–H). No dysplasia or tumors were detected in any of the bladders.

### Impaired lung morphogenesis in the UPK II-Cre;LSL-K-ras^G12D^ lungs

**Figure 3:**
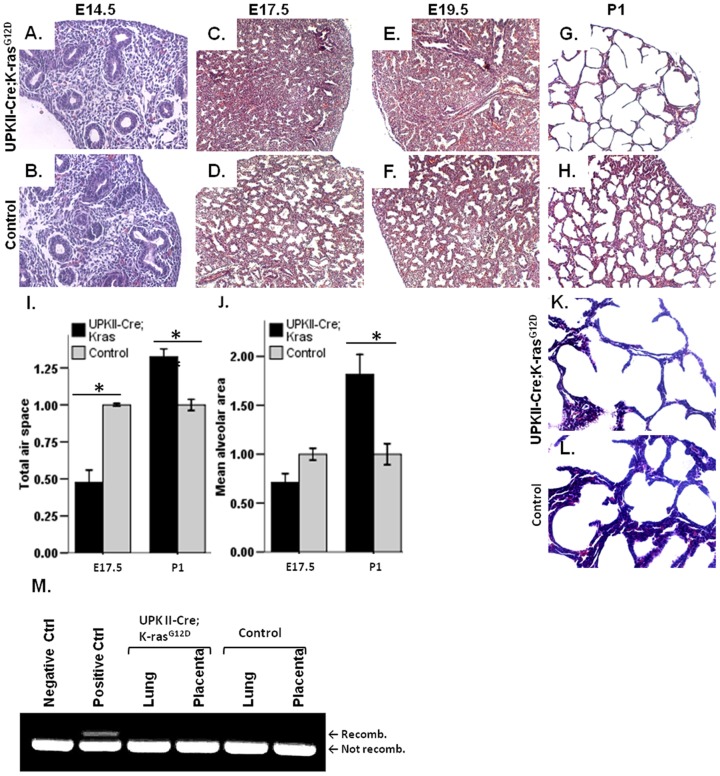
Lung phenotype of *UPK II-Cre;LSL-K-ras^G12D^* mice. A–B: Representative H&E stained sections showing normality of lung (X200) morphology in *UPK II-Cre;LSL-K-ras^G12D^* when compared with controls at E14.5 (pseudoglandular stage). **C–H**: Formation of air spaces was impaired in lungs (X100) of *UPK II-Cre;LSL-K-ras^G12D^* mice at E17.5 (**C,D**) and E19.5 (**E,F**), reflecting a defective septation process. P1 lung morphology was also different between controls and transgenic mice, which exhibited an enlargement and simplification of sacculi (**G,H**). **I–J**: Morphometric analysis of lung sections showed a decreased total air space (**I**) and mean alveolar (saccular) area (**J**) in *UPK II-Cre;LSL-K-ras^G12D^* mice at E17.5 (n = 5); the impairment of air space development led to an increased total air space area and mean alvelolar area in the early postnatal period (P1; n = 12) in transgenic mice when compared to controls; bars, SEM. **K–L**: Masson tri-chrome staining of day P1 *UPK II-Cre;LSL-K-ras^G12D^* lungs (X100), showing absence of fibrosis both in cases and controls. **M**: The direct expression of K*-ras^G12D^* was ruled out with specific PCR showing the absence of recombination between *K-ras* and the Lox sequence in lung and placenta.

Macroscopically, lungs from *UPK II-Cre;LSL-K-ras^G12D^* mice exhibited patchy hemorrhagic areas on the lung surface, with normal pleura and lobar septation. Lungs from the *UPK II-Cre;LSL-K-ras^G12D^* mice and the control mice had similar lung weights at E17.5 (*UPK II;LSL-K-ras^G12D^* vs. control: 31.8±4.32 vs 32.35±3.07 mg; *P* = 0.78), E19.5 (30.71±4.47 vs 33.89±4.54 mg; *P* = 0.10) and P1 (31.4±2.36 vs 33.01±1.26 mg; *P* = 0.52). Digestive tract, trachea and heart did not show any abnormality. Neither dilatation nor obstruction or gross abnormalities were observed in upper and lower urinary tract. Microscopically, there were no differences between *UPK II-Cre;LSL-K-ras^G12D^* and controls with regard to the pseudo-glandular phase of lung development (E14.5) (Figure 3A–B). Differences in lung morphology were not evident until E17.5, corresponding to the transition between canalicular and saccular stages or to the early saccular phase of lung morphogenesis [21]. Creation of terminal air spaces from distal airways was considerably altered in *UPK II-Cre;LSL-K-ras^G12D^* mice starting E17.5 (Figure 3C–D), and at E19.5 (Figure 3E–F). At P1, the striking impairment of septation in *UPK II-Cre;LSL-K-ras^G12D^* resulted in significantly fewer but larger terminal air spaces (sacculi) [22], which showed a simplified morphology (Figure 3G–H). Morphometry demonstrated a remarkable decrease in both total airspace and mean area of airspaces at E17.5, which indicated a possible impaired septation leading to a lower number of terminal air spaces (Figure 3I–J). A similar result was observed at E19.5 (data not shown). On day P1, increased total airspace was observed and especially in mean airspace area upon first lung insufflations (Fig. 3I–J). No inflammatory infiltrates or fibrosis were observed in P1 lung when evaluated by the Masson's tri-chrome staining (Figure 3K–L). Thus, the observed morphologic pattern suggested a normal branching of lungs with only late impairment in the process of septation. *K-ras^G12D^-Lox1A* sequence was not found in lungs of *UPK II-Cre;LSL-K-ras^G12D^* mice (Figure 3M). The lung defects in the *UPK II-Cre;LSL-K-ras^G12D^* resembled the early phase of human neonatal bronchopulmonary dysplasia, a condition in which alveolar enlargement and simplification are the key features in neonatal lungs (Figure S1, A). We did not observe fibrosis in the *UPK II-Cre;LSL-K-ras^G12D^* mice at P1, while interstitial fibrosis is noticed in human BPD only after post-natal prolonged oxygen supplementation and mechanical ventilation (Figure S1, B) [15].

### Extracellular matrix and vascular defects in the lungs from UPK II-Cre;LSL-K-ras^G12D^ mice

**Figure 4:**
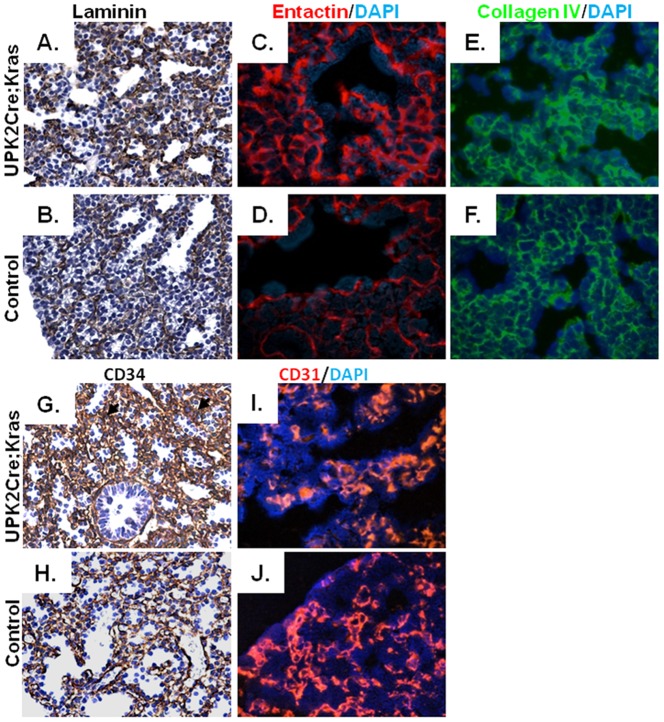
Disorganization of extracellular matrix and blood vessels in the lungs of *UPK II-Cre;LSL-K-ras^G12D^* mice. A-F: Representative images of immunohistochemistry for pan-laminin (X400) (**A,B**), and immunofluorescence for entactin (X630) (**C,D**), showed a different pattern in E17.5 lung from *UPK II-Cre;LSL-K-ras^G12D^* mice, with a less organized network and stronger expression in the stroma. Immunofluorescence for collagen IV (X630) (**E,F**) showed a similar, but less prominent, pattern. **G-J**: CD34 staining (**G,H**) and CD31 immunofluorescence (**I,J**) of lung vessels at E17.5 (X400) also exhibited a disorganized distribution in *UPK II-Cre;LSL-K-ras^G12D^* mice, with more mesenchymal vessels and a disruption of the normal subepithelial double capillary network (black arrows in **G**).

Because interactions between extracellular matrix (ECM) and epithelial/endothelial cells are critical for lung development, we examined ECM distribution in *UPK II-Cre;LSL-K-ras^G12D^* lungs. The laminin and nidogen/entactin networks, usually confined to epithelial and vascular basement membranes (BM), were altered in E17.5 lungs, with more of a stromal and diffuse distribution (Figure 4A–F). Differences in the distribution of collagen IV were not evident, but a less homogeneous staining was observed in *UPK II-Cre;LSL-K-ras^G12D^* mice (Figure 4E–F).

Due to observed laminin and nidogen (normal constituents of vascular BM) abnormalities, we next examined the integrity of the blood vessels. Immunohistochemistry analysis for CD34 (Figure 4G–H) and immunofluorescence analysis for CD31 (Figure 4I–J), revealed abnormalities in the lungs of *UPK II-Cre;LSL-K-ras^G12D^* mice at E17.5. While a normal double capillary network circling terminal spaces was observed in controls, in the lungs of the *UPK II-Cre;LSL-K-ras^G12D^* mice, capillary network distribution was diffuse and less organized, with vessels not limited to just subepithelial areas but frequently present in the stromal areas (Figure 4G-I). However, a difference in the lung VEGF expression (Western blot and immunofluorescence) was not observed between *UPK II-Cre;LSL-K-ras^G12D^* and control mice at day P1 (data not shown).

### Absence of kidney, placental or amniotic fluid abnormalities

**Figure 5:**
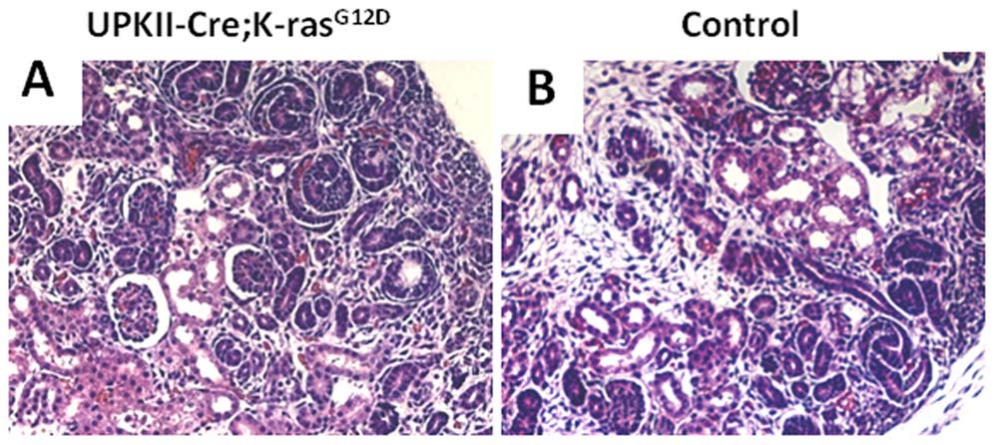
Normal kidney development in *UPK II-Cre;LSL-K-ras^G12D^* mice. A-B: H&E stained kidney sections from day E17.5 showing normal development both in *UPK II-Cre;LSL-K-ras^G12D^* mice and controls (X200).

Lung morphogenesis can be altered by amniotic fluid (AF) disorders, especially by oligohydramnios, which can be associated to kidney agenesis [23], [24]. Renal gross appearance, pyelocalicial distribution and size were normal and no agenesis or other kidney development defects were observed (Figure 5A–B). AF volume was not significantly different between *UPK II-Cre;LSL-K-ras^G12D^* and control mice at 17.5 and 14.5 gestation age (Figure S2, A). Since vessel abnormalities were present in lungs from *UPK II-Cre;LSL-K-ras^G12D^* and protein content of the AF can alter embryo development, we evaluated VEGF and sFlt1 levels in AF from gestational day 17.5. This analysis did not reveal any difference in their levels (Figure S2, C–D). Additionally, since *K-ras* codon 12 mutations may increase glycolysis [25], we measured (gestation age 17.5) AF lactate concentration. Insignificant differences were observed between *UPK II-Cre;LSL-K-ras^G12D^* (11.1±3.9 mM) and controls (10.5±1.8 mM; *P* = 0.83).

Placental disorders have also been associated with lung dysplasia [26]. Therefore, we examined placentas at gestational day 19.5. Placental weight was significantly increased in the *UPK II-Cre;LSL-K-ras^G12D^* mice (123.8±18.6 mg vs. 103±18.0 mg; *P* = 0.018) (Figure S2, B). However, placental weight on E14.5 or E17.5 was unchanged with no major defects observed in placenta at the macroscopic level or microscopically in the labyrinth and spongiotrophoblast layers of the placenta (Figure S2, E-J). The absence of the *K-ras^G12D^-Lox1A* sequence in placentas from the *UPK II-Cre;LSL-K-ras^G12D^* mice (E17.5 and 19.5) was also confirmed (Figure 3M).

### Evidence of laminin/E-cadherin fragmentation in the lungs and altered proteases activity in the amniotic fluid of UPK II-Cre;LSL-K-ras^G12D^ mice

**Figure 6:**
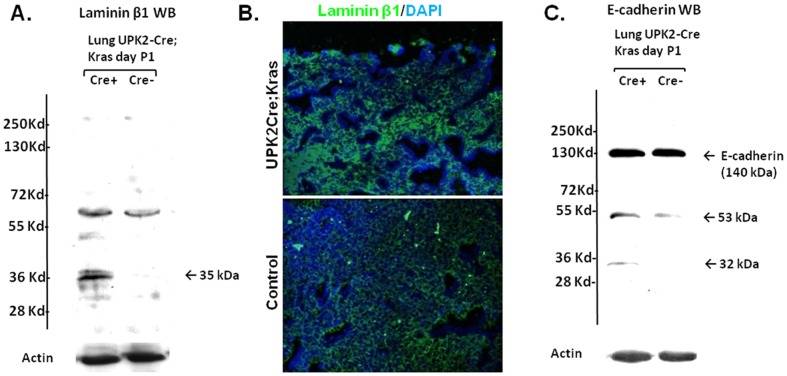
Fragmentation of ECM components in lungs of *UPK II-Cre;LSL-K-ras^G12D^* mice. A: Total protein lysates from whole lung were analyzed by Western blot for for laminin β-1 from P1 *UPK II-Cre;LSL-K-ras^G12D^* mice and control (Cre-) mice, showing an additional low molecular weight (mw) band (35 kDa) which suggest fragmentation. **B**: Representative images of immunofluorescence for laminin β-1 (X200) showed a disorganized membrane pattern in E17.5 lung from *UPK II-Cre;LSL-K-ras^G12D^* mice. **C**: WB for lung E-cadherin in P1 lung, with an increase in low mw bands. (53 and 32 KDa) also in *UPK II-Cre;LSL-K-ras^G12D^* mice.

To determine whether ECM defects in the lung was related to ECM degradation, we performed WB analysis of the ECM components in lungs of P1 *UPK II-Cre;LSL-K-ras^G12D^* mice and their littermate controls. When lung laminin β1 chain was evaluated (full length molecular weight (mw): 205 kDa), we consistently found an additional band of around 30–35 kDa, suggestive of fragmentation of laminin β1 in the lungs of *UPK II-Cre;LSL-K-ras^G12D^* mice (Figure 6A). These findings also correlated with an altered laminin β1 pattern in E17.5 lung basal membranes (Figure 6B). Immunoblotting for E-cadherin (mw: 135 kDa) clearly identified a strong 53 kDa and 32 kDa degradation band in lung samples from the *UPK II-Cre;LSL-K-ras^G12D^* mice (Figure 6C). No depletion of full-length laminin β1 or E-cadherin bands was observed, probably reflecting their higher levels of expression observed with immunohistochemistry. Since matrix metalloproteases (MMP) expression may be induced by *K-ras* mutations [27] and AF in late gestation is predominantly derived from fetal urine [28], we wondered whether *K-ras* activation in urothelium resulted in higher content of proteases in the AF, thus explaining the degradation of ECM and E-cadherin. The lungs from the *UPK II-Cre;LSL-K-ras^G12D^* mice before gestation age of E15 appeared normal and correlated with the fact that both maturation of urothelium and expression of uroplakin II occur after E15 stage of the embryo (Figure 4A–B).

We tested AF samples for gelatinolytic and caseinolytic activity (gestational days 17.5 and 19.5). Gelatin zymography of AF revealed intense MMP-2 and pro-MMP-2 bands both in *UPK II-Cre;LSL-K-ras^G12D^* and control mice. Slight differences in proteolytic activity were observed between *UPK II-Cre;LSL-K-ras^G12D^* and controls: a 20 kDa protein with gelatinolytic activity was present in the *UPK II-Cre;LSL-K-ras^G12D^* AF, which was not observed in casein zymography (Figure S3, A). In addition, two bands with caseinolytic activity of approximately 80 kDa and 70 kDa were present in AF of *UPK II-Cre;LSL-K-ras^G12D^* mice (Figure S3, B). However, Western blot analysis for MMP-1, MMP-2, MMP-3, and MT1-MMP in AF and lung did not show any differences between the two groups. Bladder expression of MMPs was also evaluated by immunofluorescence staining, but again, we did not observe differences in the expression of MMP-1, MMP-3 or MMP-13 between UPK II-Cre/K-ras^G12D^ and controls (data not shown).

## Discussion

A number of knockout and gain-of-function mouse models have provided new knowledge regarding the importance of Ras function, both during development and carcinogenesis. Here we demonstrated that specific urothelial expression of *Kras^G12D^* disrupted ECM and cell adhesion molecules in the lung and led to defective lung septation and impaired vascular patterning.

With regard to *K-ras^G12D^* expression in urothelium, the induction of urothelial proliferation is similar to that observed in other epithelia [9]. Such changes might constitute pre-neoplastic alterations, although we could not assess its future relevance due to 100% neonatal lethality of *UPK II-Cre;LSL-K-ras^G12D^* individuals. The consequence of urothelial expression of *Kras^G12D^* is the induction of a lung morphogenesis defect. We observed differences in lung morphology starting from E17.5. In that phase of lung morphogenesis (transition from canalicular to saccular stages or early saccular phase) [21] distal air spaces are generated not via branching mechanism but via septation. The finding of decreased formation of air spaces at E17.5 and E19.5 clearly correlates with a diminished number and a larger size of terminal air spaces at P1 in *UPK II-Cre;LSL-K-ras^G12D^* mice. This phenotype of normal lung branching but late impairment in the process of septation is clearly different from the early branching defects observed in lungs expressing *K-ras^G12D^* [8]. Additionally, we did not detect *K-ras^G12D^-Lox1A* sequence in lungs of *UPK II-Cre;LSL-K-ras^G12D^* mice further confirming that lung morphogenesis impairment observed in this study is not dependent on *Kras^G12D^* expression in lung tissue. The urothelial specificity of this model is further supported by our previous studies with *UPK II-SV40* mice, which displayed a constant phenotype [17] when compared to previous models using the miscloned UPK II promoter [29], suggesting that the miscloned lesion in UPKII promoter affected activity and specificity [17], [29]. These mice, with exactly the same UPK II promoter, revealed a significant cancer phenotype in urothelium with no lung defects [17]. We also excluded other potential causes of lung developmental defects, such as amniotic fluid disorders, impaired kidney morphogenesis or urinary tract obstruction. Previous reports have demonstrated that germline expression of *K-ras^G12D^* mutation induces lethal placental defects [8]. Therefore, in this study we further confirmed the absence of the *K-ras^G12D^-Lox1A* sequence in placentas from the *UPK II-Cre;LSL-K-ras^G12D^* mice. No differences in placental morphology were observed, excluding placental diseases as the cause of lung dysplasia [26].

The development of lung is a complex process in which interactions between extracellular matrix (ECM) and epithelial/endothelial cells are of crucial importance. Different mouse models have explored the role of ECM components in the fetal lung development and function, especially collagen IV, entactin and laminin [21]. Lungs from *UPK II-Cre;LSL-K-ras^G12D^* reveal a significant alteration in the ECM content, vascular basement membranes and lung capillary network.

Degradation of ECM is performed by many proteases, including the matrix metalloproteases (MMP). Oncogenic activation, and in particular *K-ras* mutations, induces MMP expression in several tumors [27] and also increase their functional levels in physiological fluids, including the urine [30]. The source of AF in the second half of gestation is predominantly via urine production by the fetus [28]. Although the fetal flow of lung liquid is predominantly outward [31], limited contact between amniotic fluid and lung fluid also exists and fetal breathing movements might introduce proteases-enriched AF to the lung. The absence of any lung defects in similar UPK II mouse models when urothelial proliferation and carcinoma are present (*UPK II-SV40* mice) [17], also suggests that mutated *K-ras* expression, and not proliferation *per se*, leads to impaired lung development in this setting. The appearance of lung changes in *UPK II-Cre;LSL-K-ras^G12D^* mice only after day E17.5, which is temporally coincident with the urothelium formation and uroplakin II expression, further supports the contribution of urine-derived AF to the observed lung morphogenetic defects. A direct degradation of ECM as a causative mechanism is suggested by the data showing fragmentation of ECM and adhesion molecules, together with the absence of inflammatory activity or fibrosis in embryo or newborn *UPK II-Cre;LSL-K-ras^G12D^* lungs, although the analysis of proteolytic activity in AF is not conclusive and further mechanistic explanations are needed.

Lungs of *UPK II-Cre;LSL-K-ras^G12D^* mice show enlargement and simplification of air spaces and a dysmorphic vascular network. This morphologic pattern resembles the pathology of early stages of human bronchopulmonary dysplasia (BPD) [15], [16]. BPD is associated with premature births (under 30 weeks) [32], corresponding to the late canalicular or early saccular stages of lung development [21], and some experimental models further support a stage-specific lesion at the saccular stage as the critical antenatal event for BPD [33]. These stages are associated with apposition of vessels and air spaces, thereby stressing the importance of the interplay between lung vascular and airway morphogenesis [34] in the pathogenesis of BPD [35], [36]. Interestingly, this stage in humans corresponds to a stage between E16.5 and post-natal day 5 in mice. It is at this stage that we observe pulmonary and vascular changes, further supporting the similarity of *UPK II-Cre;LSL-K-ras^G12D^* mice to human BPD.

Both ECM deposition and MMP activity have been shown to be critical for septation processes in lung morphogenesis [21], [22], [37], and some clinical studies suggest a role for them in BPD pathogenesis [38]. Additionally, experimental models of lung ECM disruption have revealed a connection between abnormal ECM and the defective development of airways [39], and ECM fragmentation has been shown as a mechanism for air space enlargement in other contexts such as pulmonary emphysema [40]. Our findings are also similar to previous reports that have identified a potential role of MMP-mediated E-cadherin shedding in lung morphogenesis [41]. Here we suggest the possibility that ECM disruption in the lungs of the *UPK II-Cre;LSL-K-ras^G12D^* mice could be the mechanism explaining an impaired lung septation. The vascular changes associated with human BPD are complex [42], and a role for VEGF has been speculated [32], [43]. We did not find a difference in the VEGF levels within the AF or the lung. However, vascular defects could be due to disruption of laminin and other components of vascular BM [44].

In conclusion, our results suggest that in the *UPK II-Cre;LSL-K-ras^G12D^* mice, lethal lung morphogenesis defect may be explained by altered metabolism of lung extracellular matrix and cell adhesion molecule, E-cadherin, leading to generalized ECM and vascular disruption. Our data, taken together with previous reports on the potential role of urothelium in urinary protein secretion [45], point to altered proteolytic activity in the AF as one of the causes of newborn alvealorization defects. The exact relevance of these mechanisms for human BPD pathogenesis needs more investigation.

## Supporting Information

Figure S1 **Human bronchopulmonary dysplasia (BPD).** A: A case of human bronchopulmonary dysplasia (gestational age: 24 weeks) treated with mechanical ventilation, showing enlargement and simplification of alveoli with moderate interstitial fibrosis (X100). **B**: A later stage of human BPD (gestational age: 31 weeks); only small air spaces are evident, with extensive fibrosis that impaired air exchange (X100).
(TIF)

Figure S2 **Amniotic fluid and placenta in the** ***UPK II-Cre;LSL-K-ras^G12D^*** **mice.** A: Similar AF volumes of *UPK II-Cre;LSL-K-ras^G12D^* mice and controls on gestational days 14.5 (n = 9) and 17.5 (n = 17). **B**: Increased placental weight (p = 0.018) of day 19.5 *UPK II-Cre;LSL-K-ras^G12D^* mice (n = 24) and similar weights on days 14.5 (n = 8) and 17.5 (n = 17). **C–D**: Similar AF concentration of VEGF (C; n = 4) and sFlt1 (D; n = 4). **E–J**: Low power (X25) H&E stained placenta sections from day 19.5 (**E–F**) and higher power (X200) images showing a detail of labyrinth (**G–H**) and spongiotrophoblast (**I–J**) with normal histology in both groups. Bars, SEM.
(TIF)

Figure S3 **Altered protease activity in amniotic fluid of** ***UPK II-Cre;LSL-K-ras^G12D^*** **mice.** A–B: Gelatin (**A**) and casein (**B**) zymography of days 17.5 and 19.5 amniotic fluid show additional bands of gelatinolytic (20 kDa) and caseinolytic (70–80 kDa) activity in AF of *UPK II-Cre;LSL-K-ras^G12D^* mice when compared with controls.
(TIF)
