# Supplementary figures and images for: Specific Activation of K-RasG12D Allele in the Bladder Urothelium Results in Lung Alveolar and Vascular Defects
Source: PLoS One. 2014 Apr 23;9(4):e95888. doi: 10.1371/journal.pone.0095888 (PMC3997426; doi:10.1371/journal.pone.0095888)

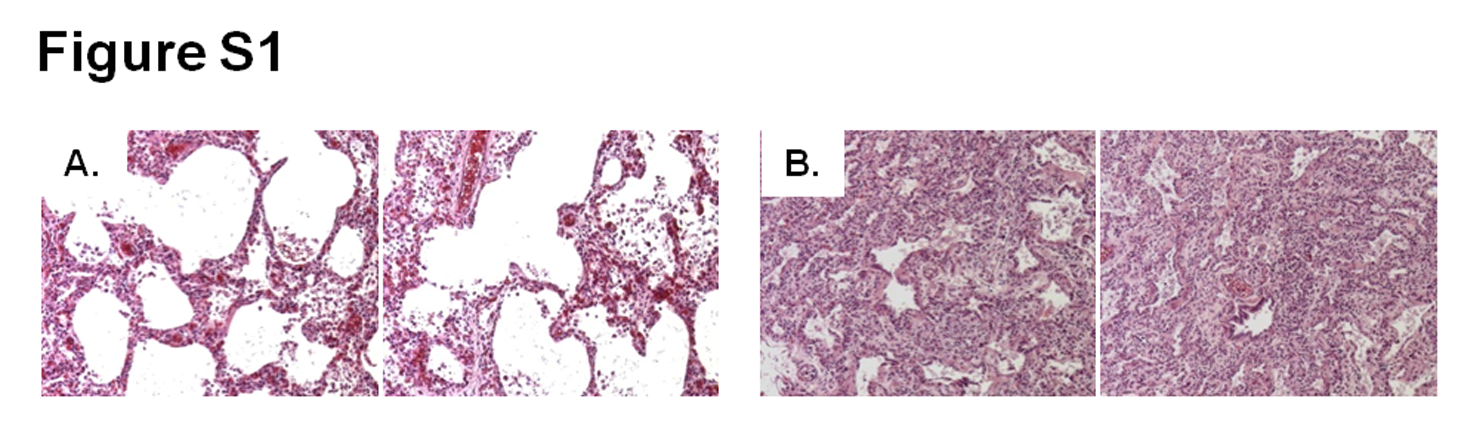

Supplement: Figure S1 — Human bronchopulmonary dysplasia (BPD). A: A case of human bronchopulmonary dysplasia (gestational age: 24 weeks) treated with mechanical ventilation, showing enlargement and simplification of alveoli with moderate interstitial fibrosis (X100). B: A later stage of human BPD (gestational age: 31 weeks); only small air spaces are evident, with extensive fibrosis that impaired air exchange (X100). (TIF) [file pone.0095888.s001.tif]

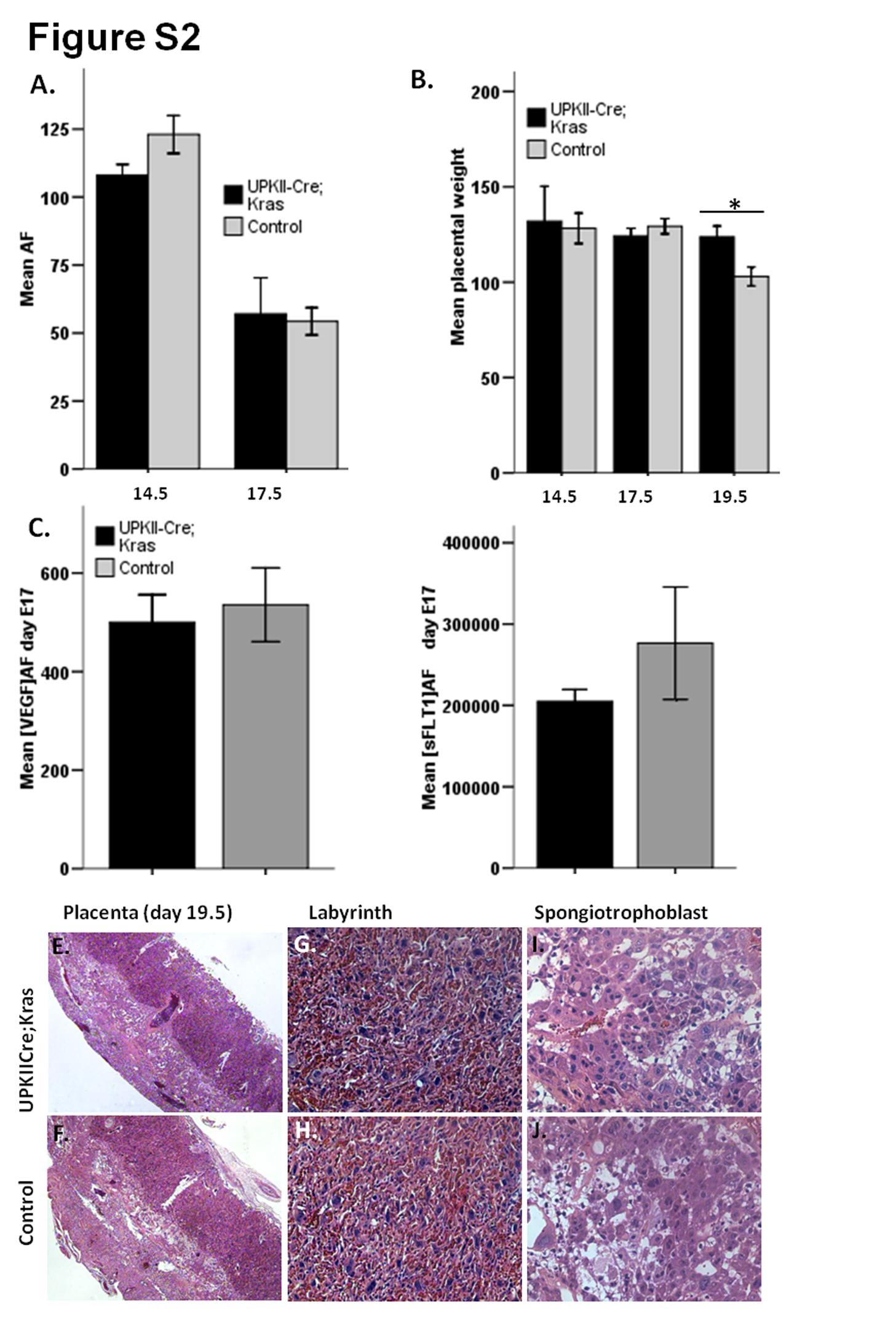

Supplement: Figure S2 — Amniotic fluid and placenta in the UPK II-Cre;LSL-K-rasG12D mice. A: Similar AF volumes of UPK II-Cre;LSL-K-rasG12D mice and controls on gestational days 14.5 (n = 9) and 17.5 (n = 17). B: Increased placental weight (p = 0.018) of day 19.5 UPK II-Cre;LSL-K-rasG12D mice (n = 24) and similar weights on days 14.5 (n = 8) and 17.5 (n = 17). C–D: Similar AF concentration of VEGF (C; n = 4) and sFlt1 (D; n = 4). E–J: Low power (X25) H&E stained placenta sections from day 19.5 (E–F) and higher power (X200) images showing a detail of labyrinth (G–H) and spongiotrophoblast (I–J) with normal histology in both groups. Bars, SEM. (TIF) [file pone.0095888.s002.tif]

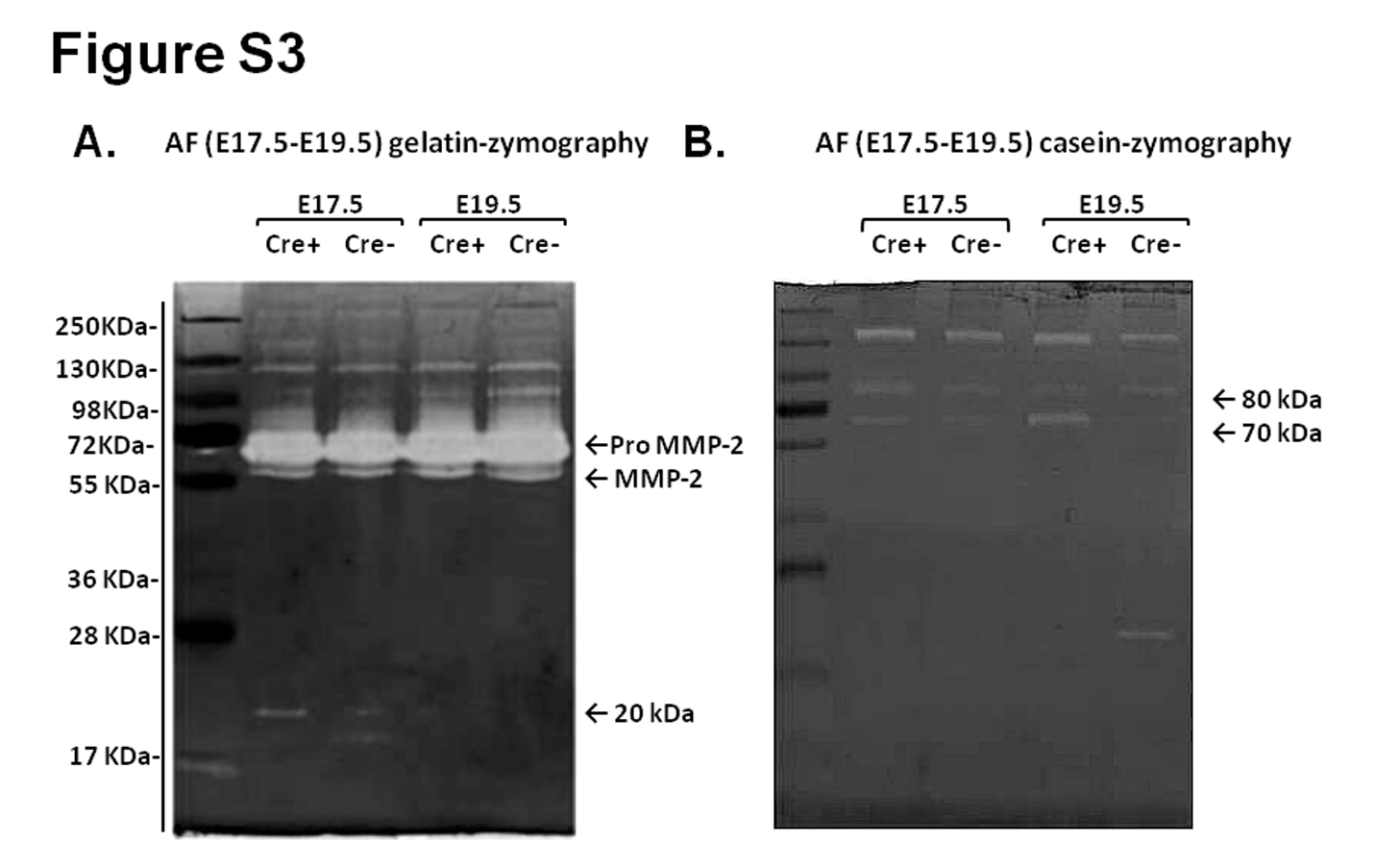

Supplement: Figure S3 — Altered protease activity in amniotic fluid of UPK II-Cre;LSL-K-rasG12D mice. A–B: Gelatin (A) and casein (B) zymography of days 17.5 and 19.5 amniotic fluid show additional bands of gelatinolytic (20 kDa) and caseinolytic (70–80 kDa) activity in AF of UPK II-Cre;LSL-K-rasG12D mice when compared with controls. (TIF) [file pone.0095888.s003.tif]
